# Clinicopathologic characteristics and treatment patterns of pelvic organ prolapse in South Korea

**DOI:** 10.11604/pamj.2019.34.14.19823

**Published:** 2019-09-06

**Authors:** Hyen Chul Jo, Jong Chul Baek, Ji Eun Park, Ji Kwon Park, In Ae Jo, Won Jun Choi, Joo Hyun Sung

**Affiliations:** 1 Department of Obstetrics and Gynecology, Gyeongsang National University Changwon Hospital, Gyeongsang National University College of Medicine, Changwon, Korea; 2 Department of Obstetrics and Gynecology, Gyeongsang National University Hospital, Gyeongsang National University College of Medicine, Jinju, Korea; 3 Department of Occupational and Environmental Medicine, Gyeongsang National University Changwon Hospital, Gyeongsang National University College of Medicine, Changwon, Korea

**Keywords:** Pelvic organ prolapse, pessary, surgery, complications, vault prolapse

## Abstract

**Introduction:**

We investigated the clinicopathologic features, method of treatment, and complications related to the conservative treatment and surgical treatment of patients with pelvic organ prolapse (POP).

**Methods:**

We retrospectively analyzed 288 patients who were diagnosed with POP from January 2007 to December 2017. The patients were divided into two groups according to the treatment method (Group A received conservative treatment and Group B received surgical treatment). The patients' clinicopathologic characteristics, treatment method, and post-treatment complications were compared between groups A and B.

**Results:**

Of the total 288 patients, 83 and 205 patients were assigned to Groups A and B, respectively. The most common symptom was a bearing-down sensation (n = 205, 71.2%), which was reported in 51 (61.4%) and 154 (75.1%) patients from Groups A and B, respectively. Among underlying diseases, hypertension was the most common in both groups (40 and 102 patients in Groups A and B, respectively). Overall, 205 patients underwent surgery, 23 underwent vaginal pessary, and 60 performed pelvic floor muscle exercises. The incidence of treatment-related complications was not significantly different between Groups A and B (13.3% vs. 17.6%, p = 0.37). Perioperative complications were noted in 20 (17.8%) patients and vault prolapse requiring subsequent surgery was noted in 16 (14.1%) patients.

**Conclusion:**

As surgical treatment is associated with recurrence and complications, conservative treatment methods can be initially considered for patients with POP. In this study, there was no difference in the incidence of complications between surgical and conservative treatments. Thus, if required, surgical treatment can be safely performed in patients with POP.

## Introduction

Pelvic organ prolapse (POP) is the descent of one or more of the female pelvic organ (vagina, uterus, bladder, rectum) into the vagina. As age increases, the prevalence of POP also increases. The cause of POP is multifactorial and established risk factors for POP include vaginal childbirth, advancing age, increasing body mass index (BMI), and prior hysterectomy [1,2]. Most patients with POP are asymptomatic and do not require treatment. Symptoms associated with POP vary and the feeling of vaginal bulging or pressure is the common and specific symptom [3,4]. Treatment should be individualized according to the severity of symptoms and patient preferences. The severity of symptoms is not consistent with the stage of prolapse [5]. Potential options for POP treatment include expectant management, conservative treatment, including vaginal pessaries or pelvic floor muscle exercises, and various methods of surgery [6]. POP is a common disease in elderly patients with comorbidities. Surgery has traditionally been associated with a recurrence or re-operation rate of up to 30% after the initial surgery therefore, conservative treatments are attempted first [7,8], but POP is a chronic problem that eventually requires surgery. The overall cumulative incidence of a subsequent surgery within five years was 7.8% for women < 65 and 9.9% for ≥ 65 years in a recent retrospective cohort study [9]. Our present study investigated the clinicopathologic characteristics, method of treatment, and post-treatment complications of POP in South Gyeongsang Province of South Korea between 2007 and 2017, using hospital-based clinical data.

## Methods

From January 2007 to December 2017, we retrospectively analyzed 288 patients with POP who visited the Obstetrics and Gynecology Departments at the Gyeongsang National University. The Korean Standard Classification of Diseases 7^th^ edition, which modified the International Statistical Classification of Diseases and Related Health Problems 10^th^ edition, was used for diagnostic specification. The definition of POP was based on the diagnostic codes for female cystocele (N811), first degree uterine prolapse (N812), complete uterovaginal prolapse (N813), uterine prolapse (N814), vaginal enterocele (N815), prolapse of the posterior vaginal wall (N816), female genital prolapse (N819), and stump prolapse (N993). The study population was divided into two groups according to method of treatment: conservative treatment (Group A) and surgical treatment (Group B). Group A was comprised of 83 patients and Group B was comprised of 205 patients. Data were grouped into the following categories: age at diagnosis, parity, BMI, underlying diseases, stage of POP at diagnosis, cystocele, rectocele, and complications after treatment. The stages of prolapse were classified by the Pelvic Organ Prolapse Quantification (POPQ) staging system.

**Statistical analyses:** nominal variables were expressed as frequency (n) and percentage (%), and continuous variables were expressed as means with standard deviations (SD). Continuous variables were compared with the use of Studen's t-tests in cases of equal variance and Mann-Whitney U tests in cases of unequal variance. Nominal variables were compared with the use of chi-squared tests in theoretical chi-squared distributions and Fisher's exact tests for samples sizes of less than five. Statistical analyses were conducted with SPSS software ver. 24.0 (IBM SPSS Inc., Chicago, IL, USA) and a p-value of less than 0.05 was considered to be significant.

## Results

Table 1 presents the general characteristics of the two study groups. There were 83 patients in Group A and 205 in Group B. The clinical characteristics of the patients with POP in Group A were as follows: mean age, 70.5 ± 10.3 years; parity, 3.9 ± 1.7; and BMI, 24.1 ± 3.6 kg/m^2^. The clinical characteristics in Group B were as follows: mean age, 68.9 ± 9.2 years; parity, 3.7 ± 1.6; and BMI, 25.1 ± 3.74 kg/m^2^. There were no significant differences between the two groups. Among underlying diseases, hypertension was the most common in both groups (40 patients in Group A and 102 patients in Group B), followed by diabetes mellitus. The most common symptom was a bearing-down sensation (n = 205, 71.2%), which was reported in 51 (61.4%) and 154 (75.1%) patients from Groups A and B, respectively. Overall, 116 (40.3%) patients had stage 2 POP, 94 (32.6%) had stage 3 POP, 17 (6.0%) had stage 1 POP, and 13 (4.5%) had stage 4 POP. Cystocele and rectocele accompanied POP in 247 (85.8%) and 171 (59.4%) patients, respectively. The incidences of cystocele and rectocele were higher in Group B than in Group A (cystocele: 72.3% vs. 91.2%, p< 0.001; rectocele: 32.5% vs. 70.2%; p < 0.001). In stage 1 patients, conservative treatment was more frequent than surgical treatment (10.8% vs. 3.9%; p = 0.049). However, in stage 2 and 3 patients, surgical treatment was more frequent than conservative treatment (stage 2: 31.3% vs. 43.9%, p = 0.049; stage 3: 19.3% vs. 38.0%, p = 0.002). Table 2 presents the treatment methods and complications in the two groups. In Group A, 23 (27.7%) patients underwent vaginal pessary and 60 (72.3%) received pelvic floor muscle training (PFMT). Perioperative complications and vault prolapse were noted in 20 (9.8%) and 16 (7.8%) patients, respectively. The incidence of treatment-related complications was not significantly different between Groups A and B (13.3% vs. 17.6%, p = 0.37).

**Table 1:** Characteristics of the two groups with conservative treatment and surgical treatment

Variables	Conservative treatment (n=83)	Surgical treatment (n=205)	*p*-value
Age	70.5±10.3	68.9±9.2	0.180*
Parity	3.9±1.7	3.7±1.6	0.276*
BMI	24.1±3.6	24.4±3.2	0.508*
**Underlying disease**			
**Cardiovascular**			
Hypertension	40 (48.2)	102 (49.8)	0.810†
Arrhythmia	5 (6.0)	7 (3.4)	0.336‡
Ischemic heart disease	4 (4.8)	13 (6.3)	0.785‡
Endocrine			
Diabetes Mellitus	14 (16.9)	28 (13.7)	0.485†
Thyroid disease	3 (3.6)	9 (4.4)	1.000‡
Cerebrovascular disease	5 (6.0)	10 (4.9)	0.771‡
**Symptom**			
Bearing down	51 (61.4)	154 (75.1)	0.020†
Protrusion mass	8 (9.6)	9 (4.4)	0.101‡
Spotting	3 (3.6)	10 (4.9)	0.763‡
Voiding difficulty	8 (9.6)	16 (7.8)	0.610†
Others	2 (2.4)	2 (1.0)	0.327‡
**Stage**			
1	9 (10.8)	8 (3.9)	0.049‡
2	26 (31.3)	90 (43.9)	0.049†
3	16 (19.3)	78 (38.0)	0.002†
4	5 (6.0)	8 (3.9)	0.531‡
Unclassified	37 (44.6)	21(10.2)	
Cystocele	60 (72.3)	187 (91.2)	<0.001†
Rectocele	27 (32.5)	144 (70.2)	<0.001†
Complication	11 (13.3)	36 (17.6)	0.370†

Values are presented as mean ± standard deviation or number (%).

* *p*-value obtained by Student’s t-test

† *p*-value obtained by chi-squared test

‡ *p*-value obtained by Fisher’s exact test.

# Stages were assessed using the Pelvic Organ Prolapse Quantitation (POP-Q) system.

**Table 2:** Method of treatment and complication of both group

	Conservative treatment (n=83)	Surgical treatment (n=205)	*p*-value
**Method of treatment**			
PFMT	60 (72.3)		
Pessary	23 (27.7)		
Complication	11 (13.3)	36 (17.6)	0.370†
Early postoperative		20 (9.8)	
Vault prolapse		16 (7.8)	

Values are presented as mean ± standard deviation or number (%).

† *p*-value obtained by chi-squared test.

PFMT, pelvic floor muscle training

## Discussion

POP is the downward descent of female pelvic organs into or through the vagina and is a common condition in women of all ages. Among patients with POP, asymptomatic POP patients do not need treatment. Patients with symptomatic POP can be managed with observation and conservative management, such as pelvic floor muscle training (PFMT), pessaries, or surgical treatment. Most patients with POP are asymptomatic. Symptomatic patients may present with a single symptom or a combination of symptoms [10]. In this study, 59 (71.0%) and 163 (79.5%) patients in Group A and Group B, respectively, presented with vaginal symptoms including, vaginal bulging or a protruding sensation. Other symptoms were urinary symptoms, such as urinary incontinence and urinary frequency or bowel symptoms, such as fecal incontinence, and the feeling of incomplete emptying. The insertion of a vaginal pessary or pelvic reconstructive surgery is one treatment method for symptomatic pelvic organ prolapse. In our study, 205 (71.2%) patients underwent surgical treatment, 60 (28.8%) patients were treated with PFMT, and a pessary was inserted in 23 (8.0%) patients. Compared with other studies [11], the proportion of patients treated with a pessary was lower in this study. The large number of patients in tertiary hospitals, like ours, referred for surgery created a selective bias, resulting in fewer patients using pessaries. All patients treated with pessaries in our study used ring pessaries. No space-filling pessaries, such as Gellhorn were used. Other studies reported successful pessary-fitting trial rates from 56 to 64% [12-14], which were higher than our overall rate of 52.18 percent (n=12). In our study, the successful pessary-fitting rate was 52.2% in Group A. The successful pessary-fitting rates were low due to the inability to use space-filling pessaries, such as Gellhorn. Jeffrey *et al.* reported that higher success rates were achieved with the use of a space-filling pessary, such as Gellhorn, and donuts in women who are unsuccessfully fitted with a support pessary (ring) [10]. We could not use these space-filling pessaries because our hospital does not supply them. However, space-filling pessaries are difficult to remove and manage by the patient alone. Those types of pessaries were not a good choice for sexually active and older patients. The common complications for discontinued pessary use after successful pessary fitting were urinary incontinence, vaginal erosion, vaginal discharge, pelvic pain, and prolapse around the pessary (aggravation of symptoms) [15]. In our study, complications after pessary use included prolapse around the pessary (n=7), infection (n=5), dysuria (n=2), and spotting (n=2). Among spotting patients, one patient was diagnosed with endometrial cancer. Women with vaginal spotting or bleeding during pessary use require further evaluation for gynecologic malignancies, such as endometrial cancer or vaginal cancer.

Unlike other gynecologic diseases, the decision regarding surgical or conservative treatment in patients with uterine prolapse, the treatment policy is influenced by the patient's preference than by the physician's opinion. The treatment should be customized according to symptom severity and patient preference. Surgical treatment is usually reserved for patients who have bothersome symptoms in at least grade 2 POP and have failed conservative treatment. A previous study reported that 12.5% of patients up to 80 years old with POP underwent surgery [16]. Surgical treatment can be performed transvaginally or transabdominally via laparotomy or laparoscopy (with or without surgical mesh). Eighty to ninety percent of surgical treatments are performed via the transvaginal approach [7,17,18]. Surgery was performed by one urogynecologist and the primary surgical procedure to repair POP was determined by the preference of the surgeon. Transvaginal surgery was performed in 194 patients (94.6%) and transabdominal surgery was performed in 11 patients (5.4%). Surgeries for POP included reconstructive procedures and concomitant hysterectomies or obliterative procedures (colpocleisis). Among the many procedures, vaginal obliteration has the highest rate of cure and the lowest of recurrence rate. However, this operation was performed only on patients without vaginal sexual activity. Three patients, aged 68, 68, and 76 underwent colpocleisis in our study. In patients who underwent surgical treatment, early postoperative complications occurred in 20 (9.8%) patients. Early postoperative complications were dysuria (n=10), urinary incontinence (n=6), fever (n=3), and fecal incontinence (n=2), and urinary frequency (n=1). Early postoperative complications were transient and reversible and mostly improved, except in one case. One patient required surgery due to newly-occurring urinary incontinence. Vault prolapse is defined by the International Continence Society as a descent of the vaginal cuff below a point that is 2 cm less than the total vaginal length above the plane of the hymen [19]. Vaginal vault prolapse is a complication following both vaginal and abdominal hysterectomies. The risk factors of vault prolapse are increasing parity, advancing age, and previous surgery due to pelvic organ support defects [20]. The prevalence of post-hysterectomy vault prolapse has been reported to range from 0.2% to 43% [21]. Recent data reported that its incidence was 11.6% following hysterectomy for prolapse and 1.8% for other pathologies [22]. A recent retrospective cohort study reported that the incidence rate of repeat surgery due to vault prolapse within five years was 7.8% in women with POP < 65 years old and 9.9% in women ≥ 65 years old [9]. A total of 16 (7.84%) patients in this study experienced vault prolapse. Our study showed a similar or lower incidence of repeat surgery compared to Western countries. Among patients who underwent surgical treatment for POP in this study, approximately 8.29% (n=17) of the patients required re-operation. Sixteen patients had vault prolapse and one patient had urinary incontinence. However, according to other reports, of those who received surgical treatment for POP, 29% will undergo another surgical treatment for POP during their life [6,16]. Long-term follow-up research is needed to determine what proportion of patients will require re-operation. This study had several limitations. First, this study was conducted in a single tertiary hospital. Therefore, there was a selection bias due to the large number of patients transferred to our hospital for surgery. Second, this study did not identify the risk factors associated with unsuccessful pessary-fitting. Third, there is a wide practice variation of surgical methods for the treatment of POP and we could not analyze the complications according to the different surgical methods. Fourth, it was difficult to show definitive results due to the small number of patients. Prospective population-based research is needed to determine the ideal treatment modality (surgical management or conservative management) for patients with POP.

## Conclusion

POP is a chronic problem that eventually requires surgery. However, patients with POP initially choose conservative treatment, which may be due to the increased risk due to the complications and recurrences that may arise from surgical treatment. This study showed no statistically significant difference between the incidence of complications in Group A and Group B (13.3% vs 17.6%, respectively; p=0.370). Surgical treatment did not increase the post-treatment rate of complications compared to conservative therapy. Surgical treatment may be effectively used to treat patients with POP.

### What is known about this topic

- Surgery incurs the risk of recurrence and complications and therefore, conservative treatment is attempted first;
- Surgical candidates with symptomatic POP are those who have failed or declined conservative management;
- Surgery has traditionally been associated with a recurrence or re-operation rate of up to 30% after the initial surgery.

### What this study adds

- Surgical treatment did not increase treatment-related complication rates compared to conservative treatment;
- Unlike earlier reports, surgical treatment can be safely performed with a re-operation incidence rate of 7.8% in women with POP.

## Competing interests

The authors declare no competing interests.
